# Decoding India’s Child Malnutrition Puzzle: A Multivariable Analysis Using a Composite Index

**DOI:** 10.3390/children11080902

**Published:** 2024-07-26

**Authors:** Gulzar Shah, Maryam Siddiqa, Padmini Shankar, Indira Karibayeva, Amber Zubair, Bushra Shah

**Affiliations:** 1 Department of Health Policy and Community Health, Jiann-Ping Hsu College of Public Health, Georgia Southern University, Statesboro, GA 30460, USA; gshah@georgiasouthern.edu (G.S.); bs06779@georgiasouthern.edu (B.S.); 2 Department of Mathematics and Statistics, International Islamic University, Islamabad 44000, Pakistan; maryam.siddiqa@iiu.edu.pk (M.S.);; 3 School of Health & Kinesiology, Georgia Southern University, Statesboro, GA 30460, USA; pshankar@georgiasouthern.edu

**Keywords:** malnutrition, stunting, wasting, underweight, composite index of anthropometric failure, children, social determinants, India

## Abstract

Background: This study examines the levels and predictors of malnutrition in Indian children under 5 years of age. Methods: Composite Index of Anthropometric Failure was applied to data from the India National Family Health Survey 2019–2021. A multivariable logistic regression model was used to assess the predictors. Results: 52.59% of children experienced anthropometric failure. Child predictors of lower malnutrition risk included female gender (adjusted odds ratio (AOR) = 0.881) and average or large size at birth (AOR = 0.729 and 0.715, respectively, compared to small size). Higher birth order increased malnutrition odds (2nd-4th: AOR = 1.211; 5th or higher: AOR = 1.449) compared to firstborn. Maternal predictors of lower malnutrition risk included age 20–34 years (AOR = 0.806), age 35–49 years (AOR = 0.714) compared to 15–19 years, normal BMI (AOR = 0.752), overweight and obese BMI (AOR = 0.504) compared to underweight, and secondary or higher education vs. no education (AOR = 0.865). Maternal predictors of higher malnutrition risk included severe anemia vs. no anemia (AOR = 1.232). Protective socioeconomic factors included middle (AOR = 0.903) and rich wealth index (AOR = 0.717) compared to poor, and toilet access (AOR = 0.803). Children’s malnutrition risk also declined with paternal education (primary: AOR = 0.901; secondary or higher: AOR = 0.822) vs. no education. Conversely, malnutrition risk increased with Hindu (AOR = 1.258) or Islam religion (AOR = 1.369) vs. other religions. Conclusions: Child malnutrition remains a critical issue in India, necessitating concerted efforts from both private and public sectors. A ‘Health in All Policies’ approach should guide public health leadership in influencing policies that impact children’s nutritional status.

## 1. Introduction

Child malnutrition continues to be a public health concern globally, impacting millions of children due to its grim consequences, including morbidity and mortality. According to the United Nations Children’s Fund (UNICEF), malnutrition in its severe form of wasting is projected to lead to the death of eight million children under the age of five in 15 countries with the worst food insecurity [1]. Nearly 40 million children in these 15 countries face severe nutrition insecurity, and almost 21 million children are severely food insecure. Although India is not among these 15 countries, malnutrition remains a challenging issue. For instance, India has the highest number of undernourished and anemic children in the world, with about one-third being underweight and more than two-thirds being anemic [2]. Paradoxically, India has one of the fastest-growing economies in the world with a 50% increase in GDP since 1991 [3].

Malnutrition in young children is a major public health concern as it increases the risk of morbidity and mortality, also impairing cognitive and physical development [4,5]. Malnutrition in childhood starts a cycle of disadvantages with negative consequences over the course of a person’s life, including a poor quality of life, maternal malnutrition, and teen marriages and pregnancies, which in turn increase the risk of offspring malnutrition [6]. The World Health Organization (WHO) has set global targets for 2025 to reduce the prevalence of stunting, wasting, anemia, and low birth weight among children [7]. These targets aim to improve the nutritional status of children worldwide and reduce the burden of malnutrition [8]. India has made some progress in reducing stunting, wasting, and low birth weight among children under five, but it is still far from the WHO global nutrition targets for 2025 [9]. India faces a triple burden of malnutrition, which includes undernutrition, micronutrient deficiencies, and overnutrition [10]. According to the Global Nutrition Report 2021, 7.7% of children are severely wasted, 19.3% are wasted, and 35.5% are stunted [11]. In addition, the progress in achieving these targets has been uneven across regions and population groups [11,12].

According to UNICEF and WHO, disparities in child malnutrition status continue to persist across the globe and in India, primarily due to children being among the least powerful constituencies in shaping political will in the allocation of resources [13]. Reports reveal that 4% of India’s GDP and 8% of its productivity is lost due to child malnutrition [14]. The lack of an adequate and sustained political focus in allocating financial resources has exacerbated hunger and malnutrition, placing India in the 102nd spot among 107 countries on the 2020 Global Hunger Index, which is calculated based on total undernourishment of the population, child stunting, wasting, and child mortality [15].

Childhood malnutrition is a complex and multifaceted problem that results from the interplay of various factors at multiple levels of influence, such as individual biological and behavioral characteristics, family socioeconomic status and food security, and community and societal structures and policies [12]. At individual and family levels, malnutrition in children is caused by a low dietary intake, poor dietary diversity, infections, and inadequate maternal nutrition [16]. The upstream influences reflected by social determinants of health (SDHs) are widely known as the predominant challenges in addressing such inequities [17,18]. Studies have shown that child undernutrition is also associated with household socioeconomic status, maternal education, sanitation, healthcare access, and gender discrimination [19]. Mothers with lower levels of education tend to have children who are average or below average in weight. In impoverished rural areas, it is reported that 40% of children suffer from being underweight [20]. Other factors affecting children’s nutritional status include caregiver knowledge, food and water availability, nutrition programs and policies, and others [21]. Environmental factors also affect nutrition status. Environmental hygiene and cleanliness impact malnutrition by affecting the development of children’s immunity and contributing to diarrhea and other illnesses [21]. There are almost 800 million people in India, with one-third of the entire population without access to sanitation facilities [8]. Children exposed to infections, parasites, or environmental toxins are more likely to suffer from malnutrition, especially wasting, which is characterized by a low weight in reference to their height [22]. Malnutrition in Indian children is associated with individual characteristics such as age, gender, and socioeconomic status. Young children need adequate essential nutrients for their growth and development, but many families cannot provide them with the required sustenance [23,24]. Female children are often neglected due to cultural bias, and preferred care is given to male offsprings [25]. Underweight women have low-birth-weight babies who inherit malnutrition [25]. Women and children also face poverty and lack basic resources.

Undernutrition is a major cause of child mortality in India, despite its fast economic growth. Many poor households still face high rates of child undernutrition, which are not explained by income, food security, or healthcare alone [24]. Recent studies suggest that interventions to reduce undernutrition should target these vulnerable groups and address the underlying inequities in education, sanitation, and healthcare that affect child nutrition [26,27]. These are the key factors that prevent India from achieving the Global Targets for 2025 set by the World Health Organization to reduce malnutrition worldwide [28].

There is a lack of comprehensive and updated research evidence concerning predictors of child malnutrition levels in India. Therefore, it is imperative to assess disparities in nutritional status among children in India and to identify the determinants and barriers to improving child nutrition. This study contributes to the body of literature on this topic by examining the levels of malnutrition and their predictors in Indian children. We pursue three research questions: (1) Which social determinants of health operationalized through household sociodemographic characteristics are associated with childhood malnutrition? (2) Which maternal characteristics are associated with childhood malnutrition? (3) Which child characteristics predict childhood malnutrition? The second point is important given that maternal characteristics such as mothers’ health, empowerment, and education can condition the predisposing factors of poor child nutrition. This study’s findings yield critical research evidence for evidence-based public health interventions and policy.

## 2. Materials and Methods

### 2.1. The Study Population and Data

Using a quantitative cross-sectional study design, this study analyzed data from India’s National Family Health Survey 2019–21 (NFHS-5) [29]. The survey covered 232,920 ever-married women from different geographical areas of India who responded to the question about the level of child malnutrition. The dataset comprised 221,263 children under five years old, but this study analyzed 210,977 survey responses by mothers about children under five years of age.

### 2.2. Outcome and Predictor Variables

This study’s dependent variable is the anthropometric indicator of malnutrition known as the composite index of anthropometric failure (CIAF), which serves as a binary indicator of anthropometric failure [30]. CIAF encompasses seven groups based on three key indicators: weight-for-age Z-score (WAZ, indicating underweight), weight-for-height Z-score (WHZ, indicating wasting), and height-for-age Z-score (HAZ, indicating stunting). These groups are delineated as follows: Group A (No failure), Group B (Wasting only), Group C (Wasting and Underweight), Group D (Wasting, Stunting, and Underweight), Group E (Stunting and Underweight), Group F (Stunting only), and Group G (Underweight only).

Conventional indices of nutritional failure, namely stunting, wasting, and underweight, classify children into various categories of malnutrition but fail to provide an estimate of the overall prevalence of malnutrition as a single measure. Therefore, to address this limitation, we employed the model developed by Peter Svedberg [31] that was later modified by Nandy et al. [32]. This model categorizes children into seven groups (A to G), enabling the calculation of the composite index of anthropometric failure (CIAF). The CIAF excludes children not experiencing anthropometric failure (group A) and includes all children who are wasted, stunted, or underweight, individually or in combination (groups B–G). Consequently, it provides a single measure for estimating the overall prevalence of malnutrition.

Observed growth performance of 210,977 children below 5 years of age was assessed in comparison with a standard performance developed by the National Centre for Health Statistics (NCHS) of the USA, which was based on growth measurements of large numbers of American children [2,4]. Deficiencies in any of the anthropometric indicators are considered an indication of malnutrition, referred to as stunting, wasting, and underweight [5]. Generally, the prevalence of undernourished children is calculated as the ratio of children who are less than 2 standard deviations of the z-score, lying below the median of the international reference population from WHO’s reference growth standards [4,7,8,9].

The predictor variables were selected based on a comprehensive review of the existing literature, encompassing nine child characteristics: child age in months, gender, birth size (height to weight ratio), birth order number (1st born, 2nd to 4th, and 5th or higher), initiation of breastfeeding, consumption of fresh milk, consumption of formula milk, breastfeeding duration, and postnatal baby checkup. Maternal characteristics comprised six factors, including the mother’s age, employment status, BMI, education level, mode of delivery (C-section), and mother’s anemia level. Societal characteristics included the following seven factors: wealth index, type of cooking fuel used, place of residency, availability of toilet facilities, water source, father’s education level, and religion.

### 2.3. Statistical Analysis

The sample characteristics were described through the presentation of a frequency-based descriptive analysis of CIAF groups. A multivariable logistic regression model was used to assess the associated risk factors of the outcome variable—CIAF—as an indicator of child malnutrition. Strength of association was measured by adjusted odd ratio (AOR), which is the ratio of the probability of an outcome in an exposed group compared to the probability of an outcome in an unexposed group, after controlling for other variables in the model. We initially included all variables in the model that were potentially associated with our outcome variable, based on the existing literature. For the selection of the best-fit model, a stepwise backward elimination method was employed as an iterative variable selection procedure. The process was repeated until only the factors significantly associated with the study outcome were selected and retained. The adequacy of the fitted CIAF model was assessed using the Hosmer–Lemeshow test and the Pearson’s chi-squared test. Additionally, to evaluate the model’s discriminatory power or its ability to distinguish between the presence and absence of CIAF anthropometric failure, a receiver operating characteristic (ROC) curve was constructed. The analysis was conducted using STATA Version 13 [33].

## 3. Results

### 3.1. Composite Index of Anthropometric Failure (CIAF) Categories in Children under Five Years Old

Table 1 provides the construction of the response variable (CIAF) via different groups. Overall, 47.41% (100,020 out of 210,977) of the children belonged to Group A (no failure). Approximately half of the children, 52.59% (110,957 out of 210,977), experienced anthropometric failure according to the CIAF indicators (Group B through G). The maximum prevalence of anthropometric failure was observed in Group F (15.56%: 32,826 out of 100,020), followed by Group E (14.50%: 30,583 out of 100,020). Conversely, Group D was observed as the least prevalent CIAF group (4.56%: 9626 out of 210,977).

### 3.2. Factors Associated with Child Malnutrition

Table 2 shows the percentage distribution of child, maternal, and societal characteristics based on the CIAF classification. Among children, the prevalence of anthropometric failure varied across age groups, with the highest rates observed in those aged 7 to 12 months (52.69%) and 49 to 60 months (49.51%). Male children experienced a 46.19% prevalence of anthropometric failure, while female children had a slightly higher rate at 48.71%. The size of a child at birth also influenced their growth, as 40.24% of small-sized newborns, 48.08% of those with average birth size, and 49.18% of large-sized newborns experienced anthropometric failure. Additionally, birth order was a factor, as first-born children had a 51.18% prevalence of anthropometric failure, while fifth or higher-born children had a lower rate of 36.60%. Among children who were immediately breastfed, 48.00% had an anthropometric failure, whereas among those who initiated it within 1 day, roughly half (50.33%) had this outcome. The distribution of children by anthropometric failure status and additional maternal and societal characteristics is also shown in Table 2.

The results of a bivariate analysis, indicating factors associated with anthropometric failure, are presented in Table S1 (a supplementary table). Nineteen factors were found to be significantly associated with the occurrence of anthropometric failure. These factors include Child Age in Months, Sex of Child, Child Birth Size, Birth Order, Initiation of Breastfeeding, Consumption of Fresh Milk, Consumption of Formula Milk, Breastfeeding Duration, Mother’s Age, Mother’s Working Status, Mother’s BMI, Mother’s Education Level, Mother’s Anemia Level, Wealth Index, Type of Place of Residence, Availability of Toilet Facilities, Water Source, Father’s Education Level, and Religion (*p* < 0.05).

Table 3 presents the multivariable logistic regression analysis results, highlighting significant factors chosen through backward stepwise elimination. These factors predict anthropometric failure based on the composite index of anthropometric failure (CIAF) in children under the age of five.

Among child characteristics, gender, child birth size, and child birth order are significantly associated with anthropometric failure, after controlling for the other variables in the model. Child characteristics associated with a lower risk of malnutrition include female gender (AOR = 0.881, 95% CI: 0.842–0.922), average birth size (AOR = 0.729, 95% CI: 0.677–0.786), and large birth size (AOR = 0.715, 95% CI: 0.655–0.780). Conversely, child characteristics associated with a higher risk of malnutrition include 2nd to 4th birth order (AOR = 1.211, 95% CI: 1.152–1.273) or having a 5th or higher birth order (AOR = 1.449, 95% CI: 1.292–1.625).

Among maternal characteristics, age, BMI, education, and anemia level are significantly associated with malnutrition, after controlling for other variables. Characteristics associated with a lower risk of malnutrition include mature maternal or age 20–34 years (AOR = 0.806, 95% CI: 0.689–0.943), age 35–49 years (AOR = 0.714, 95% CI: 0.597–0.854) rather than age 15–19, normal BMI (AOR = 0.752, 95% CI: 0.703–0.804), overweight and obese BMI (AOR = 0.504, 95% CI: 0.463–0.548), and secondary or higher education (AOR = 0.865, 95% CI: 0.806–0.927). Maternal characteristics associated with a higher risk of malnutrition include severe maternal anemia (AOR = 1.232, 95% CI: 1.059–1.432).

Societal characteristics such as wealth index, toilet facility, father’s education level, and religion are significantly associated with anthropometric failure, after controlling for other variables. Societal predictors of a lower risk of malnutrition include being in the middle wealth index (AOR = 0.903, 95% CI: 0.847–0.963) or rich wealth index (AOR = 0.717, 95% CI: 0.674–0.764), having access to toilet facilities (AOR = 0.803, 95% CI: 0.753–0.856), and the father’s level of education, whether primary (AOR = 0.901, 95% CI: 0.824–0.985) or secondary and higher (AOR = 0.822, 95% CI: 0.762–0.887). Societal factors associated with a higher risk of malnutrition include affiliation with Hindu religion (AOR = 1.258, 95% CI: 1.172–1.350) or Islam religion (AOR = 1.369, 95% CI: 1.254–1.495).

Table 4 presents an assessment of the adequacy of the fitted CIAF model. Both the Hosmer–Lemeshow test and Pearson’s chi-squared test indicate that the fitted model of nutritional failure based on CIAF is not statistically significant at *p* > 0.05, suggesting that the model is well fitted.

**Figure 1:**
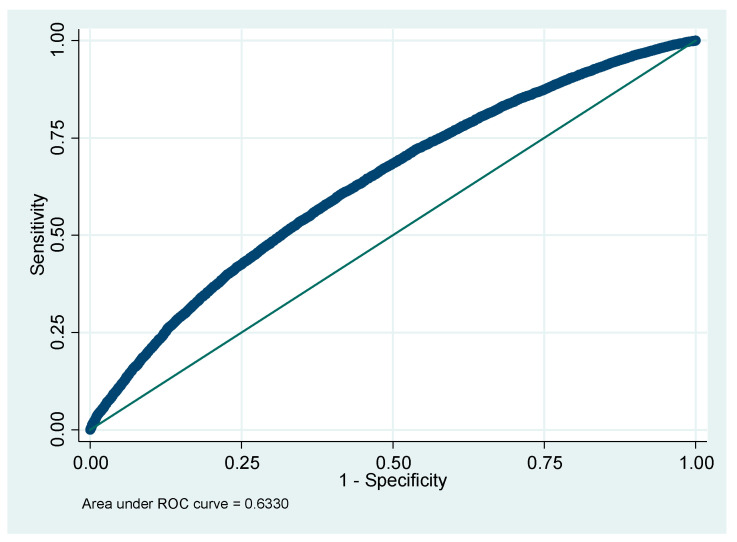
Receiver operating curve of CIAF model.

Figure 1 illustrates that the curve lies in the upper left corner, indicating that the total area under the receiver operating curve (ROC) for the CIAF model is 63.30%. Additionally, the ROC curve is closer to the upper left diagonal, suggesting better performance.

## 4. Discussion

This research study focused on addressing a critical public health issue in India, which is malnutrition among Indian children. Using India’s 2019–21 National Family Health Survey (NFHS-5), this study examined levels of malnutrition in children and evaluated the factors associated with predicting malnutrition in Indian children aiming to inform policies and interventions to address this issue. This study provided information on the anthropometric status of 210,977 children below the age of 5. The prevalence of malnutrition was assessed using the CIAF, providing a nuanced perspective on the multifaceted nature of child malnutrition.

The results of our study indicated alarming rates of child malnutrition in India, as evidenced by the CIAF indicator, which encompasses various forms of anthropometric failure. When compared to other studies utilizing the CIAF to assess malnutrition in children under five in countries such as Bangladesh, Indonesia, and Yemen, the CIAF consistently identifies more children with anthropometric failure than the individual measures of stunting, wasting, and underweight [34,35,36]. The prevalence of at least one form of anthropometric failure, as indicated by CIAF Groups B through G, highlights the widespread nature of the issue, with over half of the children (52.59%) being affected. Interestingly, our findings diverge from previously published results. The decline in underweight prevalence, observed from National Family Health Surveys (NFHSs) 3 to 4 [37,38], suggests positive strides in combating child malnutrition. Specifically, the prevalence of underweight children aged 5 and younger decreased from 42.5% during NFHS-3 (conducted in 2009–2010) to 35.1% in NFHS-4 (conducted in 2015–2016) [37,38]. However, our study highlights the paradoxical contradiction between rising malnutrition among Indian children and India’s growing global economic dominance, coupled with recent space exploration initiatives [39]. The stark contrast in resource allocation—prioritizing moon exploration over addressing food insecurity in children—serves as a poignant reminder that the true challenge lies not in resource scarcity but in strategic allocation, with a crucial focus on children’s well-being.

Our binary logistic regression analysis identified three primary predictors of anthropometric failure among the nine child characteristics initially included in the analysis: child gender, birth size, and birth order. Surprisingly, our findings did not reveal a protective role for the early initiation of breastfeeding or breastfeeding practices in general against malnutrition. This contradicts previous research that has consistently highlighted the significance of breastfeeding in promoting child health and nutrition [40,41]. It also does not align with the support from the WHO and UNICEF for initiating breastfeeding within the first hour of birth. This practice helps curb malnutrition through the protective effect of antibodies in colostrum (the first breastmilk), which shields the child against chronic diseases, promotes the child’s physical and mental development, and helps nations achieve their sustainable development goals [42,43,44]. The incongruity observed between the outcomes of our study and others may find clarification in the disparate methodologies employed to define malnutrition.

Among the six maternal characteristics examined, mothers’ age, BMI, education, and anemia emerged as significant contributors. The nutritional status of mothers plays a pivotal role in the causal pathway of child malnutrition [45]. Notably, the World Health Organization’s strategic action plan to reduce malnutrition in the Southeast Asia region reported that 36% of the population was underweight in 2016 [46]. However, our analysis of the present sample indicates that 14.09% of the mothers included in the analysis were underweight. Disparities in demographics, sample size, and geographical representation may contribute to the differences in the reported rates of underweight mothers. Nevertheless, our results also demonstrated that mothers with normal weight and overweight or obese BMIs were associated with a lower risk of anthropometric failure.

Our findings regarding the protective effect of a mother’s secondary and higher education level are consistent with the existing literature. This result aligns with Roy’s study [47], which utilized data from the National Family Health Survey 4 (2015–2016) to explore the correlation between various socioeconomic conditions and the prevalence of underweight children. Roy’s research indicated a general decrease in the risk of malnutrition with an increase in female literacy, aligning with the findings of three additional studies: Panigrahi and Das’s study (2014) in slum areas of Bhubaneswar, India; Chakraborty et al.’s study (2014) in Kolkata, West Bengal; and Victora et al.’s study (1986) in Brazilian children [48,49,50].

Among the seven societal factors examined, wealth index, availability of toilet facility, fathers’ education, and religion were significant. The lower malnutrition risk associated with educated fathers and a richer economic status aligns with the broader understanding of socioeconomic factors playing a pivotal role in child nutrition [51]. Intriguing findings emerged from Banerjee and Shirisha’s analysis of the NFHS-4, showcasing that people of a Muslim religion were in a more advantageous position overall compared to high-caste Hindus regarding wasting and low-caste Hindus across all indicators of undernutrition [52]. The authors explain that disparities observed may be attributed to certain ‘unobserved’ behavioral and cultural differences among the groups, and the authors do not provide a comparison with other religious minorities. In contrast, our study reveals a different trend, observing a higher prevalence of malnutrition in both Hindu and Muslim families compared to other religions.

Notably, although the type of cooking fuel was included in our study, it was not identified as a statistically significant predictor of children’s anthropometric failure in this analysis. However, it is crucial to acknowledge that solid fuels, such as agricultural residues, can contribute to indoor air pollution [53]. Many rural homes using agricultural residues lack proper ventilation, leading to an accumulation of these pollutants indoors [54]. Consequently, respiratory issues may impair nutrient absorption in children [55]. An analysis of NFHS-4 data revealed that the use of biomass fuels was associated with an increased risk of acute respiratory infections in children under five years of age, even after adjusting for other variables [56]. The choice of cooking fuel often reflects lower socioeconomic conditions, which can limit access to nutritious food and healthcare services [57]. In our present analysis, 8.08% of children lived in households using agricultural residues as cooking fuel, among whom nearly 40% exhibited signs of anthropometric failure. Additionally, pooled data from NFHS-1 to NFHS-4 indicate a more widespread adoption of clean fuel for cooking [58]. According to NFHS-5 results, the number of households using clean fuel increased from 16.7% (NFHS-4) to 22.6% (NFHS-5) [29]. Vir and Suri highlight that NFHS-5 data reflect an increase in women and children with access to clean fuel, which serves as a proxy indicator of women’s empowerment [59]. This underscores the importance of addressing environmental and socioeconomic factors for a comprehensive approach to improving children’s health outcomes.

Utilizing the more recent data from the NFHS-5, our study highlights the multifaceted nature of child malnutrition. An assessment of interventions aimed at mitigating maternal and child malnutrition, as outlined by Keats and colleagues, suggests employing indirect nutritional approaches to achieve nutritional advantages [60]. These approaches include strategies such as preventing malaria, providing preconception care, and promoting water, sanitation, and hygiene. Our findings are consistent with the existing literature, emphasizing the importance of a systematic and comprehensive approach to addressing socioeconomic and health-related determinants to effectively combat child malnutrition [57].

### Limitations and Future Directions

While this study took a comprehensive approach, it is important to acknowledge its limitations. The cross-sectional design restricts our ability to make causal inferences. Relying on self-reported, not independently verified data introduces potential biases inherent in self-reported surveys. Although the data for this study came from a nationally representative sample, the generalizability of some findings may require caution due to potential sampling bias, selection bias, and other limitations of such national-level surveys, which tend to miss marginalized populations.

We also acknowledge that the CIAF primarily highlights the presence of anthropometric failure but does not encompass the full spectrum of malnutrition. However, CIAF indicators are valuable for identifying populations at risk, thereby guiding further investigations and interventions. When combined with other data, these measures can more effectively inform policies and interventions. Our intention is to use these measures as part of a broader set of tools to guide interventions that holistically address child malnutrition, including its social determinants.

To bolster the strength of future research, incorporating longitudinal studies and utilizing objective measures would be beneficial. Future studies may also benefit from using mixed methods and a larger set of proximate determinants of child malnutrition, rather than relying solely on indicators collected for other purposes that may lack exhaustive information on the complex factors associated with this topic.

## 5. Conclusions

In our analysis of child malnutrition in India, several factors emerged as significant predictors. Factors associated with a lower malnutrition risk included female gender, average or large birth size, maternal age between 20–34 and 35–49 years rather than a younger age of 15 to 19, normal or overweight BMI, and higher maternal education. Socioeconomic factors such as middle-income or high-income indices rather than low-income, presence of toilet facilities, and higher paternal education also reduced the malnutrition risk in children. Conversely, a higher malnutrition risk was linked to being the 2nd to 4th child or 5th or higher, severe maternal anemia, and an affiliation with Hindu or Islam religions. Child malnutrition is a grave issue in India that warrants serious attention and concerted efforts from all agencies in the private and public sectors. In our societies, children are widely acknowledged to be the future of a nation; however, in developing countries, they continue to face inequities in public resources allocated to promote their physical and mental development, with India being on the verge of identifying as a developing country. The majority (53%) of India’s children under the age of five experiencing some form of anthropometric failure is a stark contrast to India’s status as the world’s fastest-growing economy, boasting its 2023–24 Gross Domestic Product (GDP) growth at 7.3%, with it being projected to become world’s 3rd largest economy in five years [61,62]. Such contrast in the economic ranking of the country and the unmet nutritional needs of a majority of its children lends itself to a conclusion that it is not a lack of resources that is at play but the country’s poorly strategized allocation. Such underinvestment in children may lead to growth failure and malnutrition issues, which can significantly impact their prospects. From a health equity standpoint, public health leadership in India may need to step into their role as the “Chief Health Strategist” of influencing policies in sectors affecting the social determinants of the country’s health. Through such a “Health in All Policies” approach [63], public health leadership should consider influencing all public policies that can affect the nutritional status of India’s children. The evidence from this study may provide scientific evidence for guiding interventions to holistically address child malnutrition.

## Figures and Tables

**Table 1:** Response variable (CIAF) in different groups.

Group	Description	Stunting	Wasting	Underweight	N (%)
A	No Failure	No	No	No	100,020 (47.41)
Anthropometric failure group					
B	Wasting only	No	Yes	No	13,313 (6.31)
C	Wasting and Underweight	No	Yes	Yes	14,566 (6.90)
D	Wasting, Stunting and Underweight	Yes	Yes	Yes	9626 (4.56)
E	Stunting and Underweight	Yes	No	Yes	30,583 (14.50)
F	Stunting only	Yes	No	No	32,826 (15.56)
G	Underweight only	No	No	Yes	10,043 (4.76)

**Table 2:** Percentage distribution of child, maternal, and societal characteristics based on anthropometric failure (CIAF).

Attributes	Anthropometric Failure Based on the CIAF n (%)	
	Yes	No
Child Characteristics		
Child Age in Months		
0–6	12,273 (48.85)	12,849 (51.15)
7–12	10,956 (52.69)	9837 (47.31)
13–24	18,294 (44.36)	22,946 (55.64)
25–36	19,069 (45.45)	22,887 (54.55)
37–48	19,698 (46.88)	22,321 (53.12)
49–60	19,730 (49.51)	20,117 (50.49)
Sex of child		
Male	50,377 (46.19)	58,687 (53.81)
Female	49,643 (48.71)	52,270 (51.29)
Child Birth Size		
Small	9412 (40.24)	13,979 (59.76)
Average	71,905 (48.08)	77,649 (51.92)
Large	18,703 (49.18)	19,329 (50.82)
Birth order number		
1st born	41,074 (51.18)	39,185 (48.82)
2nd–4th	54,346 (46.00)	63,803 (54.00)
5th or higher	4600 (36.60)	7969 (63.40)
Initiation of Breastfeeding		
Immediately	43,896 (48.00)	47,556 (52.00)
Within 1st hour	43,314 (46.08)	50,692 (53.92)
Within 1 day	7909 (50.33)	7805 (49.67)
Consumed Fresh Milk		
No	35,835 (45.28)	43,299 (54.72)
Yes	18,704 (48.88)	19,560 (51.12)
Consumed Formula Milk		
No	49,309 (46.34)	57,096 (53.66)
Yes	5230 (47.58)	5763 (52.42)
Breastfeeding		
No	36,472 (50.92)	35,156 (49.08)
Yes	63,548 (45.60)	75,801 (54.40)
Baby Postnatal checkup		
No	43,032 (48.09)	46,452 (51.91)
Yes	35,640 (48.41)	37,983 (51.59)
Maternal characteristics		
Mothers’ Age		
15–19	2125 (43.39)	2772 (56.61)
20–34	88,060 (47.51)	97,295 (52.49)
35–49	9835 (47.45)	10,890 (52.55)
Mothers’ Working Status		
No	11,666 (48.14)	12,566 (51.86)
Yes	3641 (45.64)	4337 (54.36)
Mothers’ BMI		
Underweight	10,836 (36.61)	18,759 (63.39)
Normal	66,349 (46.46)	76,448 (53.54)
Overweight and obese	22,402 (59.58)	15,201 (40.42)
Mothers’ Education		
No education	17,056 (37.34)	28,621 (62.66)
Primary	11,271 (41.58)	15,83 (58.42)
Secondary and higher	71,693 (51.88)	66,498 (48.12)
Delivery By C-Section		
No	77,483 (45.49)	92,840 (54.51)
Yes	22,537 (55.44)	18,117 (44.56)
Mothers’ Anemia Level		
No Anemia	42,144 (49.67)	42,700 (50.33)
Mild and moderate	54,256 (46.00)	63,696 (54.00)
Severe	2084 (41.67)	2917 (58.33)
Societal Characteristics		
Wealth Index		
Poor	42,940 (40.29)	63,626 (59.71)
Middle	20,228 (49.21)	20,879 (50.79)
Rich	36,852 (58.21)	26,452 (41.79)
Type of Cooking Fuel		
Did not cook at home	4705 (48.93)	4910 (51.07)
Electricity	1144 (52.36)	1041 (47.64)
Coal and oil	40,714 (43.34)	53,229 (56.66)
Gas	46,656 (52.91)	41,523 (47.09)
Agriculture residue	6801 (39.88)	10,254 (60.12)
Type of place of residence		
Urban	22,618 (53.44)	19,706 (46.56)
Rural	77,402 (45.89)	91,251 (54.11)
Toilet facility		
No	16,317 (37.19)	27,555 (62.81)
Yes	83,702 (50.09)	83,402 (49.91)
Water Source		
Unimproved	13,151 (47.98)	14,259 (52.02)
Improved	86,869 (47.32)	96,698 (52.68)
Fathers’ Education		
No education	1848 (36.85)	3167 (63.15)
Primary	1748 (41.78)	2436 (58.22)
Secondary and higher	11,698 (50.92)	11,276 (49.08)
Religion		
Other	14,002 (53.16)	12,339 (46.84)
Hindu	72,516 (46.90)	82,086 (53.10)
Muslim	13,502 (44.96)	16,532 (55.04)

Abbreviations: CIAF: composite index of anthropometric failure.

**Table 3:** Binary Logistic Regression Analysis of Factors Associated with Malnutrition Based on CIAF in Children under Five Years of Age.

Attributes	Anthropometric Failure on the Basis of CIAF	
	AOR	C.I
Child Characteristics		
Child Age in Months		
0–6	-	-
7–12	0.950	0.771–0.937
13–24	1.290	1.186–1.403
25–36	1.233	1.133–1.343
37–48	1.152	1.056–1.257
49–60	1.038	0.949–1.135
Sex of child		
Male	-	-
Female	0.881 *	0.842–0.922
Child Birth Size		
Small	-	-
Average	0.729 *	0.677–0.786
Large	0.715 *	0.655–0.780
Birth order number		
1st Born	-	-
2nd–4th	1.211 *	1.152–1.273
>5	1.449 *	1.292–1.625
Initiation of Breastfeeding		
Immediately	-	-
Within 1st Hour	1.000	0.937–1.067
Within 1 Day	0.946	0.837–1.070
Consumed Fresh Milk		
No	-	-
Yes	0.960	0.896–1.029
Consumed Formula Milk		
No	-	-
Yes	1.101	0.989–1.225
Breastfeeding		
No	-	-
Yes	1.173	1.112–1.237
Baby Postnatal checkup		
No	-	-
Yes	0.989	0.917–1.067
Maternal characteristics		
Mother Age		
15–19	-	-
20–34	0.806 *	0.689–0.943
35–49	0.714 *	0.597–0.854
Mother Working Status		
No	-	-
Yes	1.041	0.964–1.124
Mother BMI		
Under-weight	-	-
Normal	0.752 *	0.703–0.804
Over-weight & obese	0.504 *	0.463–0.548
Mother Education		
No-education	-	-
Primary	0.976	0.898–1.061
Secondary & higher	0.865 *	0.806–0.927
Delivery By C-Section		
No	-	-
Yes	0.878	0.827–0.933
Mother Anemia Level		
No-Anemia	-	-
Mild & Moderate	1.025	0.978–1.074
Severe	1.232 *	1.059–1.432
Societal Characteristics		
Wealth Index		
Poor	-	-
Middle	0.903 *	0.847–0.963
Rich	0.717 *	0.674–0.764
Type of Cooking Fuel		
No Cooked at Home	-	-
Electricity	0.955	0.6741–0.353
Coal & oil	1.005	0.880–1.148
Gas	1.028	0.897–1.177
Agriculture Residue	1.081	0.918–1.273
Type of place of residence		
Urban	-	-
Rural	1.032	0.948–1.125
Toilet facility		
No	-	-
Yes	0.803 *	0.753–0.856
Water Source		
Un-improved	-	-
Improved	1.016	0.885–1.165
Father Education		
No-education	-	-
Primary	0.901 *	0.824–0.985
Secondary & higher	0.822 *	0.762–0.887
Religion		
Other	-	-
Hindu	1.258 *	1.172–1.350
Muslim	1.369 *	1.254–1.495

Abbreviations: AOR: adjusted odds ratio; C.I.: confidence interval; CIAF: composite index of anthropometric failure; * designates a significant difference at *p* < 0.05.

**Table 4:** Summary of diagnostic test’s statistics for checking the model’s goodness of fit.

Tests vs. Model	CIAF $\chi^{2}$ (*p*-Values)
Pearson’s chi-squared test	17,809.18 (0.2712)
Hosmer–Lemeshow	9.21 (0.3247)

Abbreviations: CIAF: composite index of anthropometric failure.

## Data Availability

Publicly available datasets were analyzed in this study. These data can be found in World Bank’s Microdata Library at the following reference ID: IND_2019_DHS_v01_M. URL: https://microdata.worldbank.org/index.php/catalog/4482#:~:text=The%20primary%20objective%20of%20the,and%20family%20welfare%20indicators%20by (accessed on 20 October 2023).

## Supplementary Materials

The following supporting information can be downloaded at: https://www.mdpi.com/article/10.3390/children11080902/s1, Table S1. Bivariate Analysis of Factors Associated with Undernutrition Based on CIAF in Children under Five Years of Age.
