# Supplementary material for: Decoding India’s Child Malnutrition Puzzle: A Multivariable Analysis Using a Composite Index
Source: Children (Basel). 2024 Jul 26;11(8):902. doi: 10.3390/children11080902 (PMC11352507; doi:10.3390/children11080902)
Supplement: Supplementary file 1 [file children-11-00902-s001.zip › children-3036911-supplementary.pdf]

Table S1. Bivariate Analysis of Factors Associated with Undernutrition Based on CIAF in Children under Five Years of Age

**Attributes**	**Anthropometric Failure using the CIAF**	
	**OR**	**C.I**
**Child Characteristics**		
**Child Age in Months**		
0-6		
7-12	**0.857***	0.826-0.889
13-24	**1.198***	1.160-1.236
25-36	**1.146***	1.111-1.182
37-48	**1.082***	1.049-1.116
49-60	**0.973***	0.943-1.005
**Sex of Child**		
Male		
Female	**0.903***	0.888- 0.919
**Child Birth Size**		
Small		
Average	**0.727***	0.706-0.747
Large	**0.695***	0.673-0.719
**Birth order number**		
1st Born		
2nd -4th	**1.230***	1.208-1.252
>5	**1.815***	1.746-1.887
**Initiation of Breastfeeding**		
Immediately		
Within 1st Hour	**1.080***	1.060-1.100
Within 1 Day	**0.910***	0.880-0.942
**Consumed Fresh Milk**		
No		
Yes	**0.865***	0.8445-0.886
**Consumed Formula Milk**		
No		
Yes	**0.951***	0.914-0.989
**Breastfeeding**		
No		
Yes	**1.237***	1.215-1.260
**Baby Postnatal checkup**		
No		
Yes	0.987	0.968-1.006
**Maternal characteristics**		
**Mother Age**		

15-19		
20-34	**0.846***	0.799-0.896
35-49	**0.848***	0.797-0.903
**Mother Working Status**		
No		
Yes	**1.105***	1.051-1.163
**Mother BMI**		
Under-weight		
Normal	**0.665***	0.648-0.682
Over-weight & obese	**0.391***	0.379-0.404
**Mother Education**		
No-education		
Primary	**0.837***	0.812-0.863
Secondary & higher	**0.552***	0.540-0.564
**Delivery By C-Section**		
No		
Yes	0.670	0.656-0.685
**Mother Anemia Level**		
No-Anemia		
Mild & Moderate	**1.158***	1.138-1.179
Severe	**1.381***	1.303-1.463
**Societal Characteristics**		
**Wealth Index**		
Poor		
Middle	**0.696***	0.680-0.712
Rich	**0.484***	0.474-0.494
**Type of Cooking Fuel**		
No Cooked at Home		
Electricity	0.971	0.794-0.956
Coal & Oil	1.252	1.201-1.306
Gas	0.852	0.817-0.889
Agriculture Residue	1.444	1.37-1.519
**Type of place of residence**		
Urban		
Rural	**1.353***	1.324-1.382
**Toilet facility**		
No		
Yes	**0.590***	0.577-0.602
**Water Source**		
Un-improved		
Improved	**1.026***	1.000-1.053
**Father Education**		
No-education		
Primary	**0.813***	0.747-0.884
Secondary & higher	**0.562***	0.528-0.599

Religion		
Other		
Hindu	**1.284***	1.251-1.318
Muslim	**1.389***	1.344-1.436

Note: Abbreviations: C.I.: confidence interval; CIAF: Composite Index Anthropometric Failure; OR: odds ratio; bold designates a significant difference at p<0.05.
